# Endothelial cells derived from embryonic stem cells respond to cues from topographical surface patterns

**DOI:** 10.1186/1754-1611-7-18

**Published:** 2013-07-02

**Authors:** Rachel Hatano, Kevin Mercurio, Jesus Isaac Luna, Drew E Glaser, Valerie J Leppert, Kara E McCloskey

**Affiliations:** 1 School of Natural Sciences, University of California, Merced, CA, USA; 2 Graduate Program in Biological Engineering and Small-scale Technologies, University of California, Merced, CA, USA; 3 School of Engineering, University of California, P.O. Box 2039, Merced, CA 95344, USA

**Keywords:** Endothelial cells, Alignment, Contact guidance, Topography, Embryonic stem cells, Differentiation

## Abstract

The generation of micro- and nano-topography similar to those found in the extra cellular matrix of three-dimensional tissues is one technique used to recapitulate the cell-tissue physiology found in the native tissues. Despite the fact that ample studies have been conducted on the physiological significance of endothelial cells alignment parallel to shear stress, as this is the normal physiologic arrangement for healthy arterial EC, very few studies have examined the use of topographical signals to initiate endothelial cell alignment. Here, we have examined the ability for our mouse embryonic stem cell-derived endothelial cells (ESC-EC) to align on various microchip topographical systems. Briefly, we generated metal molds with ‘wrinkled’ topography using 1) 15 nm and 2) 30 nm of gold coating on the pre-strained polystryene (PS) sheets. After thermal-induced shrinkage of the PS sheets, polydimethylsiloxane (PDMS) microchips were then generated from the wrinkled molds. Using similar Shrink™-based technology, 3) larger selectively crazed acetone-etched lines in the PS sheets, and 4) fully crazed acetone-treated PS sheets of stochastic topographical morphology were also generated. The 15 nm and 30 nm gold coating generated ‘wrinkles’ of uniaxial anisotropic channels at nano-scaled widths while the crazing generated micron-sized channels. The ESC-EC were able to respond and align on the 320 nm, 510 nm, and the acetone-etched 10.5 μm channels, but not on the fully ‘crazed’ topographies. Moreover, the ESC-EC aligned most robustly on the wrinkles, and preferentially to ridge edges on the 10.5 μm-sized channels. The ability to robustly align EC on topographical surfaces enables a variety of controlled physiological studies of EC-EC and EC-ECM contact guidance, as well as having potential applications for the rapid endothelialization of stents and vascular grafts.

## Introduction

It is well documented that many cells respond to topographical surface features by changing their proliferation, adhesion, migration and/or cell orientation [1]. This interesting phenomenon is referred to as ‘contact guidance’. The physiological significance of controlling cell shape for enhancing cell-tissue function is important in a wide variety of cell types including: neurons [2-4], skeletal muscle [5], cardiac muscle [6-16], and even corneal and lens epithelial cells [17,18]. However, despite the fact that ample studies have been conducted on the physiological significance and atheroprotective benefits of endothelial cell (EC) alignment in response to shear stress (reviewed in [19]), few have incorporated the use of topographical signals to initiate EC alignment and its potential downstream physiological consequences for atheroprotection and EC repopulation after injury.

Vascular EC adhere to their underlying extracellular matrix, the basement membrane (BM), which provides a variety of biophysical and biochemical cues shown to regulate EC behavior [20,21]. The BM has many unique features, including a complex three-dimensional topography that, in turn, can influence endothelial cell function [22]. Studies also suggest that the biomechanical properties, including mechanical strength [23] and compliance [21], of the endothelial substratum influence endothelial cell behaviors. Quantitative analysis of the topographic features of aorta, carotid, saphenous, and inferior vena cava vessels isolated from the rhesus macaque indicate that vascular BM is a complex meshwork of pores and fibers mostly in the nanoscale range, but do exhibit significant differences in BM thickness and pore diameters between the different vessel tissue locations [24]. The fiber widths (i.e. ridge diameters) of the different BM isolated from a rhesus monkey remained between 25–30 nm [24] with pore sizes between 49–63 nm [25].

Mimicking the ECM topography for facilitating cell alignment has been accomplished using a variety of microfabrication approaches including: microcontact printing, abrasion, photolithography, hot embossing, electrospinning, and laser ablation [6,10,11,13,15,16], and nanofabrication approaches including: e-beam lithography andnanoimprint lithography (reviewed in [26]). Because most of these approaches are very time consuming and expensive, we used an ultra-rapid, tunable, and inexpensive non-photolithographic fabrication method to create cell culture platforms with controllable nano- and micro- scale topographical cues. The alignment grooves are created by leveraging the mismatch in stiffness between a pre-strained polymer sheet and an overlying thin metal film [27]. The wavelength of the wrinkles is tunable based upon the thickness of the metal layer on the surface of the pre-strained sheet prior to thermal exposure. When the plastic sheet retracts upon heating, the stiffer metal film buckles proportional to the thickness of the metal coating [27], causing anisotropic ‘wrinkles’ for alignment of various cell phenotypes, including cardiac cells [12]. Here, we set out to explore the ‘wrinkled’ microchip cell culture platform, in addition to two additional shrink-based platforms, for the alignment of our embryonic stem cell-derived endothelial cells (ESC-EC).

## Results

### Characterization of microchip master-mold platforms

**Figure 1:**
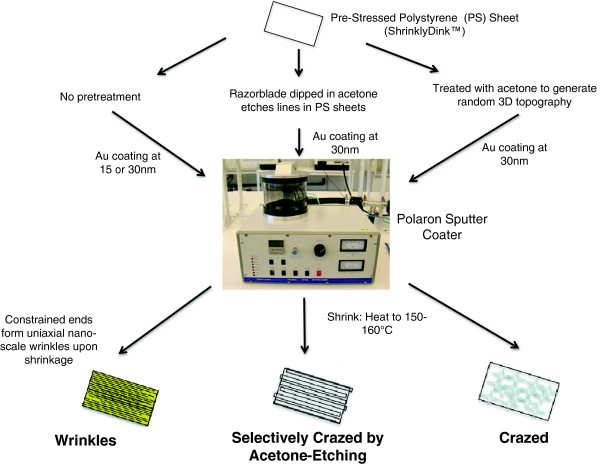
**Microchip Fabrication. ** Flowchart of three fabrication methods for the different topographies. The fabrication methods include: two different sizes of **wrinkles** that generate uniaxial nanoscale channels in the molds, selectively crazed **acetone-etched** method that generates larger micron-sized channels, and a **crazed** ‘control’ topography that does not generate uniform channels, but does exhibit random three-dimensional hills, valleys and ridges.

**Figure 2:**
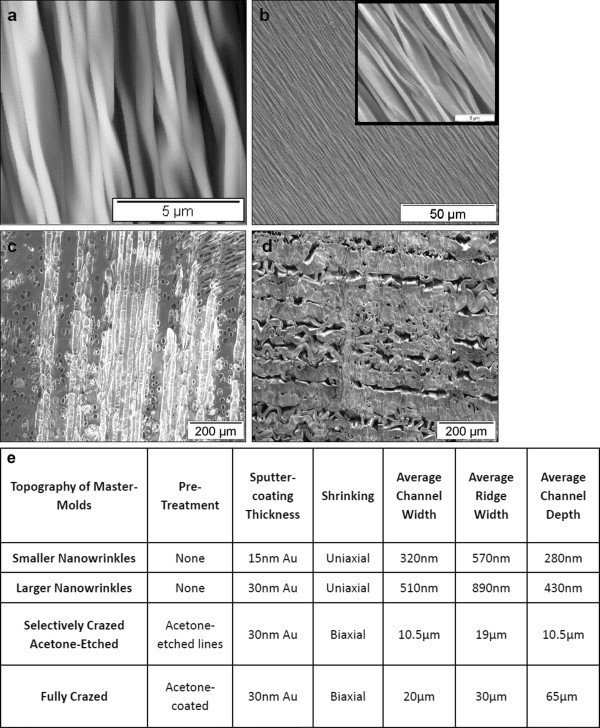
**AFM and SEM Images of the surface topography on wrinkled, selectively crazed acetone-etched channels, and crazed microchips. a)** AFM image shows the wrinkled topography developed using a 15 nm gold layer having an average wrinkle channel width of about 320 nm. Scale = 5 μm. **b)** SEM image of wrinkled topography produced using a 30 nm gold layer. Scale = 50 μm. The average wrinkle channel width is about 510 nm. Right corner: AFM image of the same, scale = 5 μm. **c)** Acetone-etched topography has an average channel width distance of about 10.5 μm. Etch lines are traveling parallel to each other after biaxial shrinking. Scale = 200 μm. **d)** Crazed topography developed after shrinking with channels about 20 μm in width. Scale = 200 μm. **e)** Table summarizing the dimensions of the four topographical surfaces.

A summary of the wrinkle methods and channel dimensions is provided in Figure 1. Nanoscale wrinkles with different dimensions were produced using two different metal deposition thicknesses: 15 nm and 30 nm gold. The 15-nm coated PS sheets generated wrinkles with average wrinkle widths of 320 nm with 280 nm peak-to-valley depths, and the 30-nm coated PS sheets generated wrinkles with average wrinkle widths of 510 nm with 430 nm peak-to-valley depths (see Figure 2). The selectively-crazed acetone-etched samples generated much larger channels of approximately 19 μm ridges and 10.5 μm valley widths (see Figure 2), but only 10.5 μm in depth. The fully-crazed topography contained an average ridge width of about 30 μm and an average valley width of about 20 μm and 65 μm in depth.

### Characterization of the ESC-derived EC

**Figure 3:**
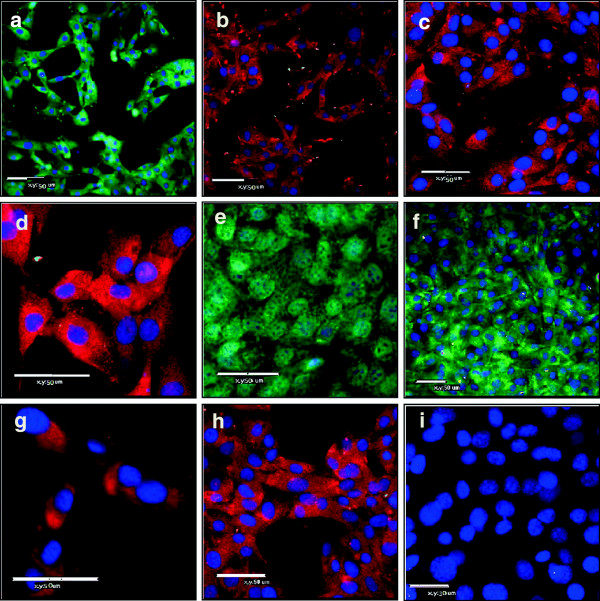
**a-i) Immunofluorescent images of ESC-EC.** These ESC-EC express endothelial markers: **(a)** Flk-1 (green), **(b)** Flt-1 (red), **(c)** Tie-1 (red), **(d)** VE-cad (red), arterial specific: **(e)** Notch-1 (green), **(f)** EphrinB2 (green), **(g)** DLL4 (red), and venous specific: **(h)** EphB4 (red), but not smooth muscle marker **(i)** calponin. All cells counterstained with DAPI (blue). Scale = 50 μm.

**Figure 4:**
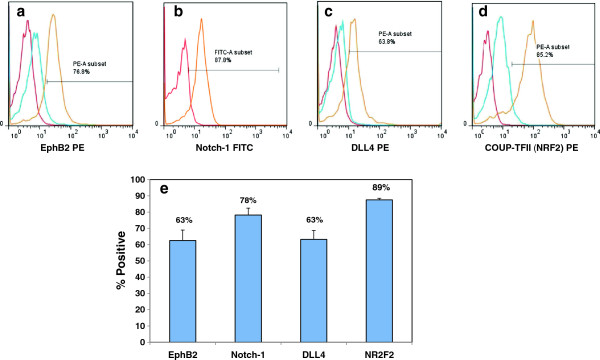
**FACS analysis of ESC-EC indicate that these cells express both arterial and venous endothelial markers.** The percent of arterial cells indicated by **(a-c)** EprinB2, intracellular Notch-1, and DLL4 staining is slightly lower than the percent of venous-specific EC **(d)** NRF2. **(e)** However, because the cultures contain over 50% of both arterial and venous phenotypes, it seems that these stem cell-derived EC cultures are retaining some plasticity regarding their arterial-venous (a-v) specification.

The cultured ESC-EC were stained for EC specific markers: Flk-1, Flt-1, Tie-1, and VE-cad, as well as arterial specific: Notch-1, EphrinB2, DLL4, and venous specific: EphB4. The smooth muscle cell (SMC) marker, calponin, was used to verify the absence on that contaminating cell types and all cells were counterstained with DAPI nuclear stain. The expression of EC and SMC markers were then analyzed by laser-scanning confocal microscopy. As seen in Figure 2a-h, the ESC-EC express all endothelial markers (Figure 3a-h), including the arterial-specific [28]: Notch-1, EphrinB2, DLL4 (Figure 3e-g) and the venous-specific [28]: EphB4 (Figure 3h), but do not express the SMC marker (Figure 3i). The percent of cells expressing the arterial-specific: EphrinB2, Notch-1, DLL4, and the venous-specific marker: COUP-TFII [29] were than quantified. The histograms of these markers (Figure 4a-d) depict the unstained cells (red), IgG control (if available; blue) and stained cells (orange). These data were than averaged (N = 3) and presented in a bar graph (Figure 4e). Based on this quantification data, it seems as if over 60% of these ESC-derived EC express arterial-specific markers, but that almost 90% also exhibit venous identity.

### Alignment of ESC-EC

**Figure 5:**
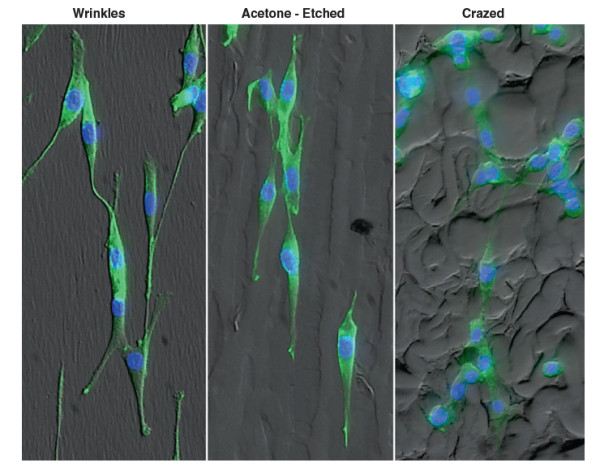
**Images of the 3 distinct topographies with cells cultured at low density.** The EC do align well with the uniaxial nanoscale wrinkles and larger acetone-etched microchannels, especially at low density. The cells also appear to adhere preferentially to the ridges compared with the valleys in the acetone-etched and crazed topographies. All cells are stained with F-actin (green) and counterstained with DAPI (blue). Magnification = 20×.

**Figure 6:**
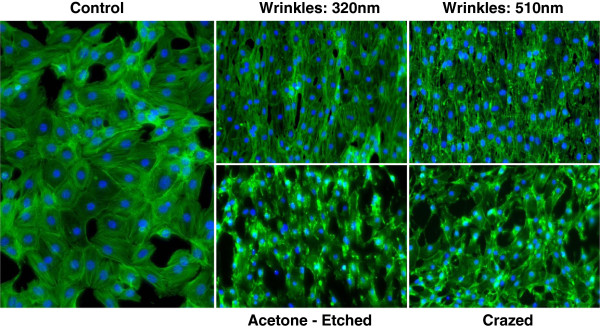
**Images of EC cultured on the 5 topographical surfaces at high density.** After 5 days of culture, the EC on the different surfaces have become confluent. Compared with the flat and crazed control surfaces, the EC align well on the wrinkles and actetone-etched uniaxial channels. All cells are stained with F-actin (green) and counterstained with DAPI (blue). Magnification = 20× (control) and 10×.

**Figure 7:**
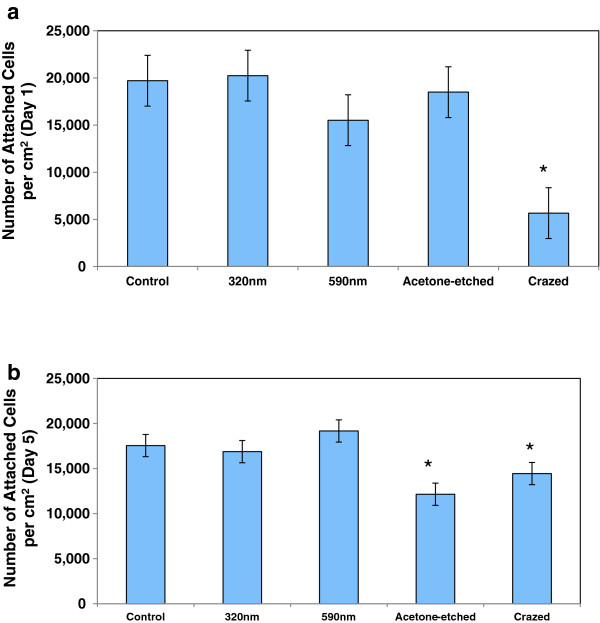
**EC adhesion on topographical surfaces.** The number of adherent EC per cm^2^ was quantified **a)** at day 1, initial adhesion, and **b)** day 5 when the cells approached confluence for that surface. The fully crazed surface significantly interfered with both the initial adhesion and the ability to reach confluence (P < 0.05) whereas, the partially crazed etched surface limited only final confluence (P < 0.05).

One day after plating ESC-EC on the different topographical surfaces (Figure 5) and staining cells for F-actin (green) and DAPI (blue), the alignment of the cells cultured on the nano-scale wrinkles and larger selectively-crazed channels is apparent. However, EC alignment and elongation are not evident on the crazed three-dimensional surface. Note that the cells also preferentially adhere to the ridges, compared with the valleys, on the selectively-crazed acetone-etched and crazed micro-scale topographies. At day 5 (Figure 6), after the ESC-EC have grown to confluence, the ESC-EC remain aligned on the wrinkle and selectively-crazed uniaxial topographies compared with the cobblestone-shaped EC grown on a flat surface (control) and the randomly crazed three-dimensional surface. The initial adhesion of the EC on the flat, nanowrinkled, and acetone-etched surfaces was significantly greater than initial adhesion of the EC on the crazed surface (Figure 7a). However, the proliferation capability of the EC was limited on the acetone-etched surface as well as the crazed surface (Figure 7b). No difference in the proliferation rates was observed (not shown).

**Figure 8:**
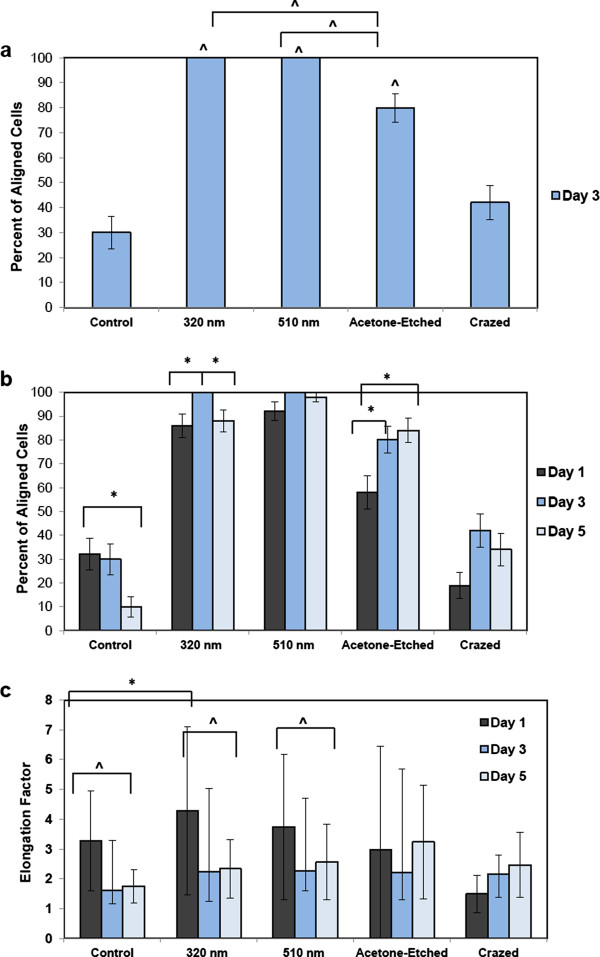
**Quantification of the aligned cells.** The aligned EC were quantified. **a)** At day 3 of culture, the cells were not yet confluent, but still had ample time to respond to topographical cues. At day 3, 100% of the cells on the two wrinkled surfaces aligned in the wrinkle direction and approximately 80% aligned on the acetone-etched surface, a much higher percentage than the control EC cultured on a flat PDMS microchip, in which only 30% aligned in the wrinkle direction ^ P-value < 0.0001. The alignment on the wrinkles is also statistically greater than the alignment seen on the larger micro-scale etched wrinkles ^, P-value < 0.0001. **b)** The EC alignment was then analyzed over time up to a confluent EC culture on day 5. For the small 390 nm wrinkles, a statistically significant peak in alignment is seen at day 3 compared with day 1 and day 5. For the large micro-channels in the acetone-etched surfaces, the percent of EC aligning on the channels increase over time, whereas the percent of aligned cells decrease for the flat control surface, * P-value < 0.01. **c)** The average elongation factors were also calculated for all topographical surfaces and time points. The greatest EC elongation was seen in the 390 nm wrinkled surface at day 1, and was a statistically significant increase compared with the flat control surface, * P-value < 0.05. Moreover, as the cells became more confluent, they began to lose some of their elongation, ^ P-value < 0.0001.

The percent of ESC-EC aligning on the different topographies was then quantified. Based on the criterion that the cells were “aligned” if their long-axis was oriented within ±30° with the channel direction, 100% of the cells cultured on the 2 sizes of nano-scale wrinkles were aligned by day 3, and close to 80% of the cells on selectively-crazed channels (Figure 8a). These were all statistically significant compared with the control and crazed topographies. The percent of cells aligned on the nano-scale wrinkles was also statistically greater than the acetone-etched micro-channels, suggesting that the multiple nano-scale cues provide stronger (i.e. more) signals for the cells to align. The alignment on the small nano-scale wrinkles also increases from day 1 to day 3, but then is reduced when the cells become confluent by day 5 (Figure 8b), whereas the percent of aligned cells on the acetone-etched surfaces continue to increase from day 1, to day 3, to day 5.

We also calculated the elongation factors for these cells (N = 50; Figure 8c). On day 1, EC cultured on the smaller, 320 nm, nano-scale wrinkles exhibited the greatest elongation, and this was statistically greater compared with the control EC. Moreover, the EC cultured on the flat control surface and both wrinkled surfaces exhibited the greatest elongation on day 1 with decreased elongation by day 3 and 5. Conversely, the elongation factors for cells on the selectively-crazed channels remained similar over time, and were not significantly elongated compared with control EC. It is important to note that EC cultured on flat surfaces (control) also elongate more at subconfluence, but appear more cobblestone-shaped at confluence (Additional file 1: Figure S1). Based on the comparison with control cells, we know that there is a reduction in elongation from subconfluent to confluent cultures, and that the wrinkles aid the elongation of confluent EC.

## Discussion

The stained cells depicted in Figure 2a-h, combined with previously published characterization studies [30-33], illustrate that the ESC-derived EC are composed of largely pure populations of EC-specific cells. However, in our experience, the arterial or venous (a-v) identity of the ESC-EC can vary somewhat from isolation-to-isolation, with most cells predominantly expressing venous EC markers. Therefore, we first set out to characterize the a-v identity of the ESC-EC used for these alignment studies. Ephrin-B2 and EphB4 mark arterial and venous EC, respectively [34] while Notch1 and DLL4 are also considered markers of arterial EC [28] and have been shown to play a role in arterial specification from stem cells [35]. The data indicate that over 50% of the ESC-EC must be expressing both phenotypes simultaneously, as greater than 60% express arterial EC markers, while 90% express the venous EC marker. Because reciprocal signaling between Ephrin-B2 positive arteries and EphB4 positive veins is crucial for morphogenesis of the capillary beds, this data may indicate that our ESC-EC more closely resemble the capillary EC subphenotype compared with an arterial or venous phenotype.

After plating the ESC-EC on gelatin-coated PDMS microchips with the nano- and micro-scale topographies, the ESC-EC align with both 320 nm and 510 nm ‘wrinkles’ as well as on the ridges (Figures 5 and 6) of the larger 10.5 μm channels formed in the selectively-crazed microchips, with the greatest cell alignment seen on the nano-sized wrinkles compared with the larger channels (Figure 8a). This data agrees closely with one other study that compared four human vascular endothelial cell-types from large and small diameter vessels [20]. This study found that orientation and alignment of human umbilical vein endothelial cells (HUVEC) was the most pronounced on 800 nm anisotropically ordered ridges, but the EC isolated from larger vessels preferred the larger (1200 nm-4000 nm) topographies [20].

Although significant differences in the ESC-EC adhesion and proliferation cultured on the nano-scale wrinkles compared with flat surfaces were not observed, the larger crazed surfaces did limit both adhesion (fully crazed only) and the ability for the cells to proliferate (partially crazed and fully crazed surfaces). This data agrees with a study that found no difference in cell adhesion of adipose-derived stem cells compared with flat substrates [36]. Another study that examined pyramid-shapped features, ranging from 50 nm-2 μm in height, also found that the fewer cells adhered to the rough surfaces and that micro-scaled pyramids also hindered migration [37], like the reduced adhesion we observed on our crazed surface.

It was also seen that as the ESC-EC are cultured on the nano-scale wrinkles, the percent of aligned cells and elongation factor is slightly reduced at day 3 and 5 as the cells also become more confluent (Figure 8b). This is consistent with the elongated (although not directionally oriented) morphology of subconfluent EC and the cobblestone-shaped morophlogy of EC cultured on flat surfaces (Additional file 1: Figure S1). We expect that the cell-to-cell adhesions via cadherins are beginning to compete with cell-substrate integrin attachment sites as the cell cultures become confluent. Interestingly, this phenomenon of reduced alignment at confluence is not observed in EC cultured on the larger micro-scale channels. The width of an EC that has elongated along a ridge-edge, D_min_, is smaller than the 10.5 μm channel width, and therefore, the cell-to-cell interactions across the large channel widths would be more difficult to establish. Moreover, because the ESC-EC, as well as other EC [38], preferentially adhere and align on the ridges of the topographical features, the percent of aligned ESC-EC on the larger micron-sized channels actually continued to increase (Figure 8b) with time, but probably are less likely to bridge the large channels gaps for generating confluence. Other studies have also confirmed that micron-scale topographies actually interfere with cell migration and have proposed a 2-step model of cell adhesion [37] suggesting that the surface topography guides initial cell contact, but that the engagement of the cell surface receptors is controlled by the surface chemistry (i.e. ligand density).

In addition to using this microchip topographical platform to study the physiologic response to surface-induced cell alignment, the technology also has potential applications in regenerative medicine. Due to the highly thrombotic surface of the disrupted endothelium following coronary angioplasty (stent insertion), approaches for enhancing endothelial cell adhesion via cell elongation are being explored including surface modification for generating micro- and nano- structured surfaces [39]. Moreover, because shear stress signaling on confluent and aligned endothelium is known to impart athero-protective benefits, it is likely that the EC alignment could be advantageous in both cell adhesion and anti-thrombosis in the absence of other mechanical forces.

## Conclusions

This work describes the use of distinct 3D topographical cell culture platforms for aligning ESC-derived EC. Consistent with the small anisotropic nano-scale fiber sizes in the BM of vessels, the ESC-EC align most robustly on the anisotropic nano-scale wrinkles. On the larger micron-sized channels, the ESC-EC preferred to align with the ridge edges of the channels and do not seem to form the lateral cell-cell borders that lead to reduced elongation of the EC at confluence. Characterization of the ESC-EC showed that they express a number of EC markers, including both arterial- and venous-specific markers. The dual positive a-v identity may be an indication that these cells are consistent with a microvascular EC subphenotype. The ESC-EC aligning most robustly on the smaller nano-scale topology is also consistent with a micro-vessel subphenotype.

## Methods

### Generation of microchips with nano- and micro-scale topography

Metal wrinkles were fabricated as previously reported and described in detail [27]. Briefly (Figure 1a), gold was deposited by sputter coating (SEM Sputter Coater; Polaron) at various thicknesses. After deposition, the pre-strained polystyrene (PS) sheets (ShinkyDinks, Inc.) were induced to thermally shrink by heating to a temperature of 150–160°C. When the pre-strained polystyrene was exposed to temperatures above glass transition (T_g_ for polystyrene ~ 105 Celsius), the polystyrene shrinks and contracts. When constrained at opposite ends, the buckling of the surface layer produces the wrinkled topography. The metal coating treatments allow some control over wrinkle dimensions: 15 nm to 90 nm coating correlates with average wrinkle widths ranging from 800 nm to 1 μm, increasing proportionally with coating thickness [27]. These ‘wrinkled’ metal surfaces serve as master molds for generating PDMS microchip cell culture platforms. A 10:1 ratio of PDMS and curing agent (Sylgard 184 Silicon Elastomer Kit, Dow Corning) was poured on the metal mold and set to cure at 75°C. The chips were sterilized following standard procedures and then coated with 0.5% gelatin and seeded with cells. For this study, we generated four different surface topographies to attain different wrinkle wavelength dimensions. The first two use the standard procedure described above, depositing 15-nm and 30-nm of gold onto the pre-strained PS sheets. The third topography, called selectively crazed acetone-etched, uses acetone with a razorblade to selectively etch ‘lines’ onto the pre-strained polystyrene sheets. The width of the etch lines is controlled based on the thickness of the razor blade edge and the depth of the channel is determined by exposure to the solvent prior to thermal shrinking. The final topography, called ‘fully crazed’ is generated after the entire surface of the PS sheet is treated with acetone, then coated with gold metal and thermally shrunk by heating to 150–160°C without constraining edges of the PS sheet. The crazing of polymer surfaces to produce this type of topography has been well-explained in the literature [40].

### Characterization of microchip master-mold platforms

The depth and width features of the nano-scale wrinkles were characterized using the Atomic Force Microscope (AFM) while the dimensions in the acetone-etched and crazed topographies were characterized using Scanning Electron Microscopy (SEM). Cross sections of the platforms were used to verify the height features in the crazed topographies. The AFM was operated in tapping mode and the total scan size was 10 μm × 10μm. Once images were captured, analysis was conducted using AnalySIS Pro software to determine the range of dimensions.

### ESC cell culture

Mouse D3-ESC (American Type Culture Collection, Manassas, VA) were initially maintained on irradiated or Mitomycin C-treated (Sigma) mouse embryonic fibroblast feeder layers in Knockout Dulbecco’s Modified Eagle Medium (KO-DMEM; Gibco) containing 15% ES Cell Qualified Fetal Bovine Serum (Gibco), 5% Knockout Serum Replacement (Gibco), 1,000 units per ml of leukemia inhibitory factor (ESGRO; Chemicon International) and 5 × 10^-5^ M β-mercaptoethanol. Cells were then cultured on 0.1% gelatin (no feeders) for one week before switching to differentiation conditions.

### EC derivation from ESC

The EC used in these studies were derived from mouse ESC using previously published protocols [30-33,41]. Briefly, initial induction of EC required 4 days of culture on collagen type IV-coated dishes in media containing FBS and without leukemia inhibitory factor (LIF). Differentiation medium consisted of 93% alpha-Minimal Essential Medium, 5% Fetal Bovine Serum, 1% Penicillin/Streptomycin, 1% L-Glutamine, and 5 × 10^-5^ M β-mercaptoethanol. The cells expressing Flk-1 were then sorted using magnetic cell sorting (MACS®, Miltenyi Biotech) and allowed to grow for one week on collagen type-IV coated dishes. After one week, the Flk1^+^ positive cells exhibited 2 phenotypes; elongated smooth muscle morphology or cobblestone-like endothelial morphology. The cells exhibiting endothelial morphology were manually selected and fed endothelial cell medium (EGM-2 medium supplemented with EGM-2 Bullet Kit; Clonetics - 10 ml FBS, 0.2 ml hydrocortisone, 2 ml hFGF-β, 0.5 ml VEGF, 0.5 ml R3-IGF-1, 0.5 ml ascorbic acid, 0.5 ml hEGF, 0.5 ml GA-1000, 0.5 ml heparin – plus 5 × 10^-5^ M β-mercaptoethanol, and an extra 50 ng/ml of recombinant human VEGF; VEGF_165_, R&D Systems). Methods consistently yield 25 population doublings at >95% purity [31].

### ESC-EC characterization: confocal imaging

Cells were fixed with 4% paraformaldehyde (PFA) and permeabilized with 0.7% Triton 100× in buffer containing 0.5% bovine serum albumin and 5% donkey serum. Primary rabbit antibodies against EphrinB2 (Santa Cruz Biotech) and calponin (Santa Cruz Biotech), as well as, goat antibodies against Flt-1 (BD Pharmingen), Tie-1 (SC Biotech), EphB4 (Santa Cruz Biotech), and VE-cad (Santa Cruz Biotech), were added at concentrations of 1 μg total per sample and refrigerated overnight. The next day, the cells were rinsed before adding FITC anti-rabbit (Fitzgerald) and PE anti-goat (Santa Cruz Biotech) antibodies which were incubated for an additional 2 hours before imaging. Directly-conjugated antibodies for Flk-1 FITC (BD Pharmingen), Notch-1 FITC (Abcam) as well as PE-conjugated antibodies against Delta-like Ligand 4 (DLL4; Biolegend) were also incubated overnight. Cells were imaged using a laser-scanning confocal microscope (Technical Instruments).

### ESC-EC characterization: FACS analysis

Cells were removed with Cell Dissociation Buffer (Invitrogen), fixed with 4% paraformaldehyde (PFA) and permeabilized with 0.7% Triton 100× in buffer with 0.5% bovine serum albumin. Rabbit antibodies against EphrinB2 (Santa Cruz Biotech) and Coup-TFII (NR2F2; Abcam), Notch-1 (Abcam), their IgG controls, and PE conjugated DLL4 (Biolegend) were added at concentrations of 1 μg total per sample and refrigerated overnight. The next day, the cells were rinsed with PBS before adding FITC anti-rabbit (Fitzgerald) or PE anti-goat (Santa Cruz Biotech) antibodies which were incubated for an additional 2 hours before FACS analysis.

### ESC-EC on nano- and micro-scale topographies

Each of the PDMS microchips was coated with 0.5% gelatin and then plated with 20,000 ESC-EC per cm^2^. At day 1, 3 and 5, the ESC-EC were fixed with 4% paraformaldahyde, stained for F-actin (Atto 488 Phalloidin, Sigma) and counterstained with DAPI. Samples were mounted on a cover glass using mounting medium (Vector Laboratories) and imaged with an inverted fluorescent microscope (Nikon Eclipse TE2000-U) and digital camera (Photometrics Coolsnap). ESC-EC were defined as “aligned” if their major axes are within ± 30° with respect to the wrinkle or channel direction (the “aligned” direction was randomly chosen for control and crazed surfaces with no distinct direction). Based on this criterion, the percentage of cells on the channeled surface was quantified. All comparisons for statistical significance were conducted using a student’s *T*-test using the mean percentage and standard error of the mean (SEM) for N = 50 cells. The elongation factor (EF) was also calculated by the ratio of the maximal diameter (D_max_), length, to the minimal diameter (D_min_), or length-to-width ratio of the cell (EF = D_max_/D_min_) [42]. All comparisons for statistical significance of EF were conducted using a Student’s *t*-test using the mean EF and standard deviation (SD) for N = 50 cells.

## Competing interests

The authors declare that they have no competing interests.

## Author information

Kara E McCloskey: https://eng.ucmerced.edu/people/kmccloskey/KaraMcCloskey

## Authors’ contributions

The first author, RH, along with DG, characterized the ESC-derived EC used in this the study and initiated all cell culture experiments and data analysis of images. The second author, KM generated and characterized the cell culture surface materials. JL collected the fluorescent images. The principle investigators, VL and KMcC, led the study: reviewed all experimental procedures, data collection methods, and data analyses. All authors read and approved the final manuscript.

## Supplementary Material

Additional file 1: Figure S1 Images of ESC-EC on flat surfaces*.* The ESC-EC exhibit increased elongation in subconfluent cultures (left) compared with confluent cultures (right). Click here for file
