# Supplementary figures and images for: Endothelial cells derived from embryonic stem cells respond to cues from topographical surface patterns
Source: J Biol Eng. 2013 Jul 2;7:18. doi: 10.1186/1754-1611-7-18 (PMC3711924; doi:10.1186/1754-1611-7-18)

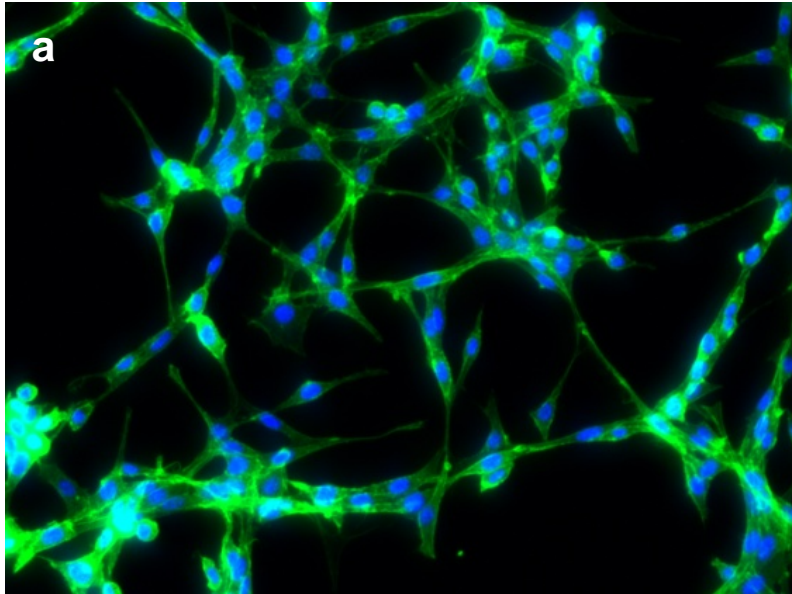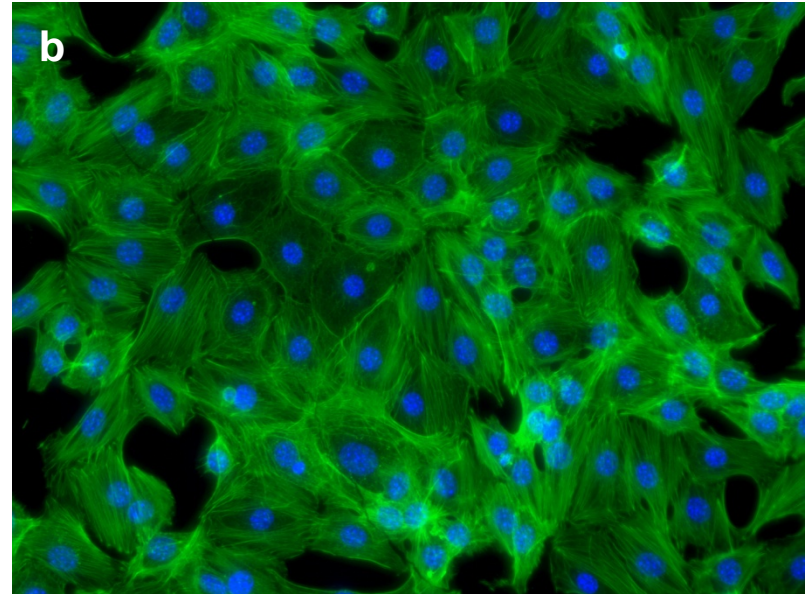

Figure S1

Supplement: Additional file 1: Figure S1 — Images of ESC-EC on flat surfaces. The ESC-EC exhibit increased elongation in subconfluent cultures (left) compared with confluent cultures (right). [file 1754-1611-7-18-S1.pdf]
